# Molecular identification and lipolytic potential of filamentous fungi isolated from residual cooking oil

**DOI:** 10.3897/BDJ.12.e113698

**Published:** 2024-02-05

**Authors:** Elvia G Gómez-Vázquez, Yazmin Sánchez Roque, Guillermo R Ibáñez-Duharte, Miguel A Canseco-Pérez, Ana G Zenteno-Carballo, Roberto Berrones-Hernández, Yolanda C Pérez-Luna

**Affiliations:** 1 Universidad de Ciencias y Artes de Chiapas, Tuxtla Gutiérrez, Mexico Universidad de Ciencias y Artes de Chiapas Tuxtla Gutiérrez Mexico; 2 Universidad Politécnica de Chiapas, Suchiapa, Mexico Universidad Politécnica de Chiapas Suchiapa Mexico

**Keywords:** isolation, morphology, taxonomy, homology, phylogenetics, filamentous fungi

## Abstract

Filamentous fungi, microorganisms that develop and are located in different habitats, are considered important producers of enzymes and metabolites with potential for the biotechnology industry. The objective of this work was to isolate and identify filamentous fungi that grow in used oil. Two fungal species were characterised through their morphology and molecular identification. The DNA of each extracted strain was amplified by PCR using primers ITS1 and ITS4, obtaining sequences that were later in GenBank (NCBI). A white coloured strain (HB) with a cottony, white, hyaline morphology and irregular borders was observed; so too, a brown colony (HC) with a sandy surface, a well-defined border of beige colour in early growth until it became a dark brown colour. The identity result by homology of the sequences in the BLASTn database was 100% and 99.55%, indicating that they correspond to *Cladosporiumtenuissimum* and *Fomitopsismeliae*, respectively. Finally, the results in lipolytic activity show greater potential for *Fomitopsismeliae* with 0.61 U/l in residual oil. Thus, it is important to highlight the potential of this type of waste to favour the prospection of microorganisms for a sustainable alternative for future studies of biological conversion.

## Introduction

Filamentous fungi are eukaryotic organisms that develop in different environmental as food, agro-industrial waste, soil and plants (Powers-Fletcher et al. 2016). These organic residues can be fats and oils that originate in kitchens or in the food industry. However, these wastes are the cause of soil and river contamination and are also producers of toxins that cause damage to health and affect the quality of life by generating bad odours in the environment. That is why these products, with the help of fungi isolated from the same environment, allow the possibility of a biological conversion to new products with added value (Kumar and Negi 2015,Liu et al. 2021,Mohammadi-Nasrabadi et al. 2019,Orjuela and Clark 2020).

Fungi are considered saprophytes and colonise substrates by growing highly polar multicellular cells called hyphae (Cairns et al. 2019,Füting et al. 2021) whose growth occurs septately or continuously in the cell wall, these significant differences in structure favour the classification of fungi (Powers-Fletcher et al. 2016). On the other hand, the reproduction of fungi can be asexual and sexual. The asexual type produces conidia or distributed spores without nuclear fusion, while the sexual type involves the union of two compatible haploid nuclei (Cole 1996). Therefore, the organisational forms of the spores determine the taxonomic categories (phylum) (Powers-Fletcher et al. 2016).

From a social and economic perspective, these microorganisms play an important role in the pharmaceutical, agricultural, food, detergent, leather, paper industries and biofuel (El-Sawah et al. 2021,Hyde et al. 2019,KC et al. 2020,Meyer 2021, Molina-Gutiérrez et al. 2021,Singh et al. 2021), for the generation of hydrolytic enzymes, which can be used in novel biotechnological processes (Ramos Sanchez 2015,Syedd-León et al. 2022).

In this sense, lipases constitute a promising alternative to replace chemical catalysis in biodiesel synthesis. In particular, fungal lipases are the most widely used due to their lower cost and higher operational stability. These enzymes are widely distributed in nature and, from a physiological point of view, their function is to catalyse the catabolism reactions of triglycerides into fatty acids and glycerol, occupying a central place in lipid metabolism.

Another interesting quality of filamentous fungi is their ability to degrade complex organic matter, transforming it into new biodegradable components to be absorbed by the soil and terrestrial flora, generating an evolutionary cycle of recycling (Téllez Vargas et al. 2017,Mancilla et al. 2020). In addition, they are known to secrete high levels of hydrolytic enzymes and secondary metabolites that have biotechnological applications (Füting et al. 2021,Siddiqui 2016), due to the natural metabolic activity of filamentous fungi that allows optimising cell factories producing specific metabolites, for development and scaling. of bioprocesses (Meyer 2021). Therefore, this work aims to characterise and evaluate the lipolytic potential of two filamentous fungi isolated from used cooking oils, with the purpose of identifying new strains from organic environments (waste), so that, in future research, they can be evaluated with residual substrates (recycled oils) from where they originate for various biotechnological applications.

## Material and methods

### Study site

The study was carried out in the Biotechnology Laboratory of the Polytechnic University of Chiapas, Mexico, located in Suchiapa, Chiapas, located at 16° 37' north latitude and between 93° 06' west longitude. The altitude is 530 m above sea level. The climatic conditions of this site correspond to warm subhumid with an average annual temperature of 16-24°C (Anonymous 2023).

### Sample collection

Samples of recycled vegetable oil from food frying (inexpensive kitchens and restaurants) in the City of Tuxtla Gutiérrez, Chiapas, Mexico, were collected and deposited in previously sterilised glass jars with a capacity of 5 litres.

### Raw material characterisation (recycled oil)

To characterise the recycled oil samples, the physicochemical properties of relative density, viscosity, humidity and volatile matter were evaluated. The relative density was determined according to the NMX-F-075-SCFI-2012 standard, while the humidity and volatile matter with the NMX-F-211-SCFI-2012 standard and, for the parameter of viscosity, a Cannon Fenske 150 series viscometer was used.

### Isolation of microorganisms and selection of fungi

The microorganisms were isolated from waste samples of used vegetable oils from kitchens where food is prepared. To do this, the surface spread method was used on PDA agar plates (4 g potato extract, 20 g dextrose and 15 g agar) at 28-30°C for 7 days. The strains were subsequently inoculated successively to have selective cultures, in a PDA solid culture medium supplemented with 0.1 g of chloramphenicol and incubated at 28-30°C for 7 days. Each isolate was preserved in triplicate in vials with PDB medium (4 g potato starch and 20 g dextrose) and 40% glycerol, these being stored at -20°C (Mendes et al. 2019).

### Morphological identification

For the evaluation of the morphological characteristics at the macro- and microscopic level of the selected strains, a comparison was made by means of determined taxonomic keys (family/genus) of studied strains, through the capture of images taken directly on the PDA plates and in samples previously prepared in cotton blue solution to locate mycelium, fruiting bodies, conidia arrangement, amongst others (Guarro et al. 1999,Money 2016,Naranjo‐Ortiz and Gabaldón 2019).

### Molecular identification

For the molecular identification of the selected fungi, genetic sequencing was considered, for which genomic DNA was extracted from the strains obtained by the CTAB method and amplified with ITS1 (5´-TCCGTAGGTGAACCTGCGG-3´) / ITS4 (5´-TCCTCCGCTTATTGATATGC-3´) using the PCR (Polymerase Chain Reaction) technique, which was carried out in a 20 μl reaction volume that included the following components: 1 μl of template DNA (100 ng), 0.5 μl of a mixture of 2.5 mM dNTPs, 2 μl of 10X PCR reaction buffer, 1.2 μl of a 50 mM MgCl_2_ solution, 0.5 μl of a 10 μM oligonucleotide solution, 0.2 μl of Phusion™ High- Fidelity DNA Polymerase (2 U/µl) (ThermoFisher Scientific) and milli-Q water (for a final volume of 20 µl). PCR was carried out in a thermocycler (Bio-Rad) with the following conditions: a denaturation cycle at 95°C for 7 min, a second denaturation step at 95°C for 1 min, 35 amplification cycles with a alignment stage for 30 s at 60°C, an elongation stage of 1 min at 72°C and a final extension stage at 72°C for 3 min. PCR products were separated by 0.8% agarose gel electrophoresis at 70 volts for 1 h in 1x TAE buffer. The amplified fragments of each strain correspond to the internal transcribed spacers 1 and 2 (ITS), which were purified with the commercial kit Zymoclean Gel DNA Recovery Kit (Zymo Research, Orange, CA, USA), run in a 2% agarose gel and were subjected to sequencing at the IPICYT National Laboratory for Agricultural, Medical and Environmental Biotechnology (LANBAMA) in San Luis Potosí (Raeder and Broda 1985,Tel-zur et al. 1999).

### Identity by homology and phylogenetic relationship

The obtained sequences that encode the internal transcribed spacer (ITS) regions of both fungi were used to identify their homologues using the NCBI Blastn tool (Altschul et al. 1990). A multiple alignment was carried out using the MEGA 11 software (Tamura et al. 2021) with the Clustal W algorithm, the five sequences with the highest homology for each amplicon were taken and fungal sequences previously reported by Bensch et al. (2012) and by Liu et al. (2021), for HCITS4 and HBITS4, respectively, were identified. The phylogenetic analysis was performed using the Maximun Likelihood method with 1000 bootstraps, using the IQ-Tree software (Trifinopoulos et al. 2016). The resulting phylogenetic trees were edited using I-Tol software (Letunic and Bork 2011).

### Evaluation of lipolytic activity of selected fungi through the qualitative determination of rhodamine B

To detect lipolytic activity of the selected strains, it was determined through the detection of orange fluorescence haloes around the colony, under UV light at 350 nm where the selected strains were seeded on PDA plates supplemented with 1% (p/p) of olive oil (w/w) or 1% (w/w) of recycled oil and 0.01% (w/w) Rhodamine B, incubated at 25-30°C for 7 days (Kumar et al. 2012, Mendes et al. 2019); finally, the presence or absence (+ or -) of formation by the fluorescent haloes was validated.

### Determination of enzyme activity (lipolytic)

In order to carry out this test, the crude extract of the strains was obtained. Subsequently, the lipase activity assay was carried out with p-nitrophenyl-palmitate (p-NPP) as a substrate. The cell-free supernatant (0.1 ml) was added to 0.9 ml of the substrate mixture containing solution A (3 mg pNPP in 1 ml isopropanol) and solution B (10 mg gum arabic and 40 mg of Triton-X in 9 ml of 50 mM Tris-HCl buffer pH 8). Absorbance was measured spectrophotometrically at 410 nm after 30 min incubation at 37°C in a shaking water bath. One unit of activity (U) was defined as the amount of enzyme that releases 1 μmol of p-nitrophenol/min under the assay conditions. All assays were performed in triplicate and mean values were calculated. Protein concentration was determined using bovine serum albumin (BSA), as described by Bradford (Eko Sukohidayat et al. 2018,Pacheco et al. 2015).

## Results and discussion

### Sample collection

The used oil samples were of vegetable origin. This waste is generated in significant quantities by society in general; it is estimated that in some parts of the world a person generates 173 and 290 kg/year in Europe (Mtui 2012), while in Mexico, the average is 56 kg/year (Caldeira et al. 2017,Patinha et al. 2017). It is important to mention that 1 litre of oil can contaminate more than a million litres of water (Kumar and Negi 2015) when discarded in effluents.

The effective collection and controlled disposal of recycled oils is of great importance at an economic level, due to its viability for energy production (De Feo et al. 2020). To contribute to the reduction of this waste, currently, recycled oils are used as raw material for their transformation through the production of low value-added products such as biofuels, soaps, animal feed, amongst others (Orjuela and Clark 2020).

### Raw material characterisation (recycled oil)

The used oil of vegetable origin presented a dark brown colouration and a rancid odour. These two qualities represent the oxidation of a recycled cooking oil (Liu et al. 2022,Totani et al. 2012). These oils may be corn, sunflower, palm, coconut, peanut, soybean, cottonseed, canola and safflower which are used for frying food items. They commonly undergo transformations through hydrolysis, polymerisation and thermal degradation processes (Adhikesavan et al. 2022). In addition, physicochemical properties were evaluated (Table 1) that allowed defining the sample under study, because some of the most notable changes in the oil when frying foods are viscosity, density and humidity (Adhikesavan et al. 2022,Choe and Min 2007).

The results of the density of the oil coincide with those reported by Tomazzoni et al. (2013) who mention that vegetable oils have a relative density between 0.840 and 0.960; since it is less than that of water, all oils float in it and remain on the surface and, therefore, it is important to mention that the values are associated with temperature (15°C), because, as the temperature increases, the oil expands, and, therefore, its density decreases (NMX-F-075-SCFI-2012). On the other hand, the viscosity present in these residual oils is classified with a low viscosity index, since it is below those established by NOM-116-SCFI-2018 and ASTM D-445 (high index greater than 80 mm^2^/s), so they will experience a decrease in their viscosity with respect to the increase in temperature (Esfe et al. 2018). Finally, the determination of moisture and volatile matter is particularly important for the useful life of oils. The useful life is the period during which the oil can maintain its sensory, chemical, physical and nutritional properties if it is stored in adequate conditions; therefore, the values obtained are below the ideal values (1-2%) according to Ordoñez et al. (2013), the foregoing due to the inadequate forms of collection and storage of residual cooking oils (Orjuela and Clark 2020). However, this evaluated physicochemical characteristic generates an ideal environment for the propagation of microorganisms (Liu et al. 2022), especially those of high resistance to stress caused by temperatures, viscosity and volatile material; in this sense, filamentous fungi lead this group of microorganisms (Kumar and Negi 2015,Syedd-León et al. 2022), since they grow under the physicochemical characteristics identified in the oils collected in this research work.

### Isolation of microorganisms and selection of fungi

**Figure 1.**
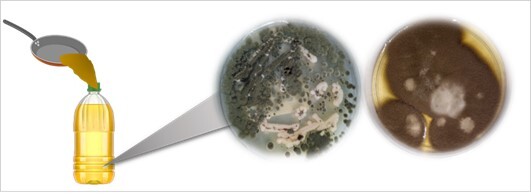
Microorganisms isolated from used cooking oil.

Subsequently, for the isolation of microorganisms, the PDA medium was used and the boxes inoculated with recycled oil were incubated for 7 days at 28-30°C; after this time, the presence of filamentous fungi was detected, demonstrating growth through the difference in colouration. A fast-growing brown fungus (HC) and a white fungus (HB) were visualised (Fig. 1).

These results demonstrate great physiological adaptability and metabolic capacity (Peraza-Jiménez et al. 2021) of filamentous fungi to grow in nutrients with these characteristics. Various studies have proven that fungi could take up carbon sources for their potential growth. These studies promote the significance of biovalorisation of the oily materials as a substrate for fungal growth, especially since the demands and usage of fatty acids are increasing (Papanikolaou et al. 2011,Hamdi et al. 2022). They are heterotrophs and require organic materials that they use as an energy source and as carbon skeletons for cell synthesis. They are widely distributed in nature, being found in very diverse habitats; hence, their frequent appearance as spoilers in food, as well as being recognised for their resistance to living conditions and high duplication rates, unlike bacteria (Powers-Fletcher et al. 2016,Téllez Vargas et al. 2017,Füting et al. 2021). Therefore, the isolation of native microorganisms from organic matter gives them certain characteristics to express their potential for the production of enzymes and to be able to be used as an alternative in problem areas of residual origin (Chaudhary et al. 2022, Ghosh et al. 2023, Richards and Talbot 2018, Talukdar et al. 2022).

### Morphological identification

**Figure 2.**
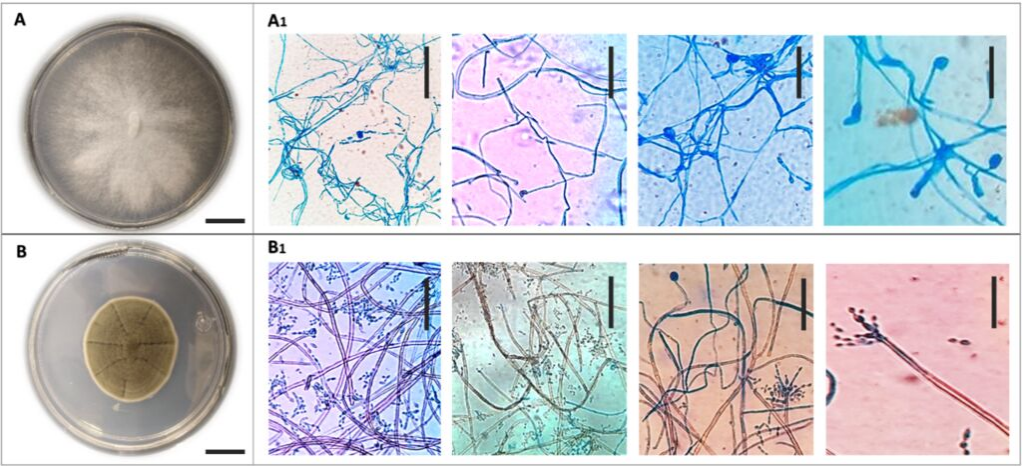
Macroscopic and microscopic morphology of HB (**A**) and HC (**B**) in PDA medium at 7 days of growth. **A, B** Colonial morphology (A1 and B1). Microscopic visualisation (40x). Scale bars: A, B = 1 cm; A1, B 1 = 10 µm.

The white fungus (HB strain) presented a hyaline white cottony morphology with irregular borders (Fig. 2A), while the brown fungus (HC strain) appeared in dark green colonies with a sandy surface with a well-defined beige border in early growth until it was left in a brown colour, somewhat rough-walled, thin and subhyaline, but sometimes becoming thick-walled with age lightly pigmented to dark brown (Fig. 2B). The microscopic characteristics of the HB strain show that they present continuous mycelium and endogenous spores (sporangiospores). These characteristics correspond to zygomycete fungi that have a thallus of coenocytic hyphae in which the nuclei are haploid when the organism is in the vegetative stage (Meyer et al. 2020) and, for the HC strain, septate mycelia and endogenous spores (ascospores) were visualized, most of the known fungi belonging to this Phylum Ascomycota, which is characterised by the formation of an ascus (plural, asci), a sac-like structure that contains haploid spores (Naranjo‐Ortiz and Gabaldón 2019). (Bensch et al. 2012) mention that *Cladosporium* colonies are greyish-brown to dark brown, effuse, confluent, thickly felted to villose. Mycelium immersed; hyphae branched, 2–7 μm wide, septate, with constrictions at the septa, hyphal cells sometimes irregularly swollen, sometimes irregularly lobed, subhyaline to pale olivaceous-brown, hyphae giving rise to conidiophores often darker, medium to dark brown and walls somewhat thickened.

The morphological analysis of the white fungus demonstrates similarity with *Fomitopsis*. The genus is characterised by sessile to effused basidiomas, a perennial growth habit, white to tan or pink pore surface with small, regular pores, generative hyphae attached in a di- or trimitiquiphal system and thin hyaline walls and smooth basidiospores (Patel et al. 2021).

### Molecular identification

For the molecular characterisation of the fungi, fragments of the internal transcribed spacer (ITS) of the genomic sequences of said strains were identified, obtaining the genomic DNA of each one and later the product was amplified by PCR with the primers ITS1 and ITS4. Fragments of 500-650 bp in size were obtained in both strains. This result confirms the advantage of using the internal transcribed spacer (ITS) region as a standard marker, because it is located between the fungal 18S rRNA, 5.8S rRNA and 28S genes and is also used to analyse the genetic diversity and taxonomic identification of fungal species, based on this genomic region (Badotti et al. 2017,Kim 2011).

### Identity by homology and phylogenetic relationship

The result of identity by homology of the sequences in the BLASTn database was determined by two parameters: the E-value whose value should be close to a score of 0.0 and the percentage of identity, which was 100% and 99.55%, which correspond to *Cladosporiumtenuissimum* and *Fomitopsismeliae*, respectively (Table 2).

The homology with the sequence with accession number OQ780870 indicated that the genus of the white fungus corresponds to *Fomitopsismeliae*, suggesting that it belongs to the kingdom fungi in the division of Basidiomycota (basidiomycetes). While the sequence accession number OQ780869 of the brown fungus belongs to *Cladosporiumtenuissimum*, suggesting that the genus corresponds to *Cladosporium* and the kingdom fungi, in the division of Ascomycota (ascomycetes). The literature describes important contributions of filamentous fungi with biotechnological potential, related to the Ascomycota phylum that were isolated from soils contaminated with grease or oily residues (*Geotrichum* sp., *Penicillium* sp., *Aspergillusniger*, *Aspergillus* sp., *Penicilliumchrysogenum*, *Yarrowialipolytica*, *Alternariatenuissima*, *Fusariumoxysporum*) and Mucoromycota (*Rhizomucormiehei*, *Mucor* sp.), while, in the Basidiomycota division, there is little evidence of these microorganisms (Adamczak and Bednarski 2004,Carvalho et al. 2005, Cesário et al. 2021,Kumar and Negi 2015,Hita et al. 2009).These microorganisms confirm their identity through the morphological and molecular information evaluated, demonstrating differences in the phylum.

The phylogenetic analysis of each strain was carried out using the Maximun Likelihood method with 1000 bootstraps; it was obtained through the analysis of five sequences with the highest homology for each amplicon, using the IQ-Tree software (Trifinopoulos et al. 2016).

**Figure 3.**
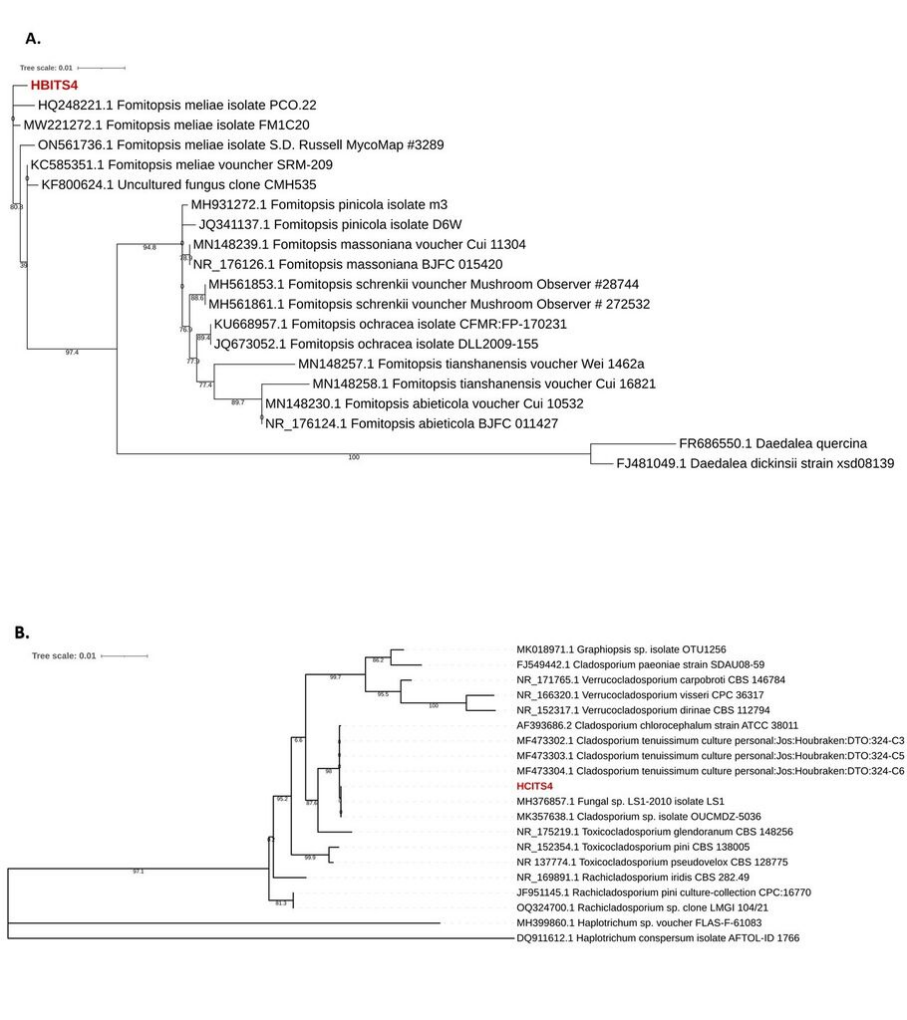
Phylogenetic tree of *Fomitopsismeliae* (**A**) and phylogenetic tree of *Cladosporiumtenuissimum*, both made in MEGA 11 (**B**).

In Figure 3, two phylogenetic trees can be visualised: (A) they correspond to *Fomitopsismeliae* (HBITS4) and (B) to *Cladosporiumtenuissimum* (HCITS4). The HBITS4 strain was analysed with the five homologues closest to the amplicon and was also compared with other *Fomitopsis* taxa that were reviewed in a phylogenetic analysis inferred from the sequence data of the internal transcribed spacer (ITS) regions that represent 11 taxa belonging to the Phylo Basidiomycota (Liu et al. 2021), resulting in the sequence being associated with the taxon of the species meliae (Fig. 3A). While the HCITS4 strain was also compared with five homologous sequences identified by the NCBI Blastn tool (Altschul et al. 1990) and other sequences belonging to *Cladosporium*, these sequences were selected from a genus analysis of hyphomycetes *Cladosporiums*., the differences evaluated between the clades, based on their phylogeny, systematic and ecological characteristics (Bensch et al. 2012), demonstrating that they are related to the genus *Cladosporium* and belonging to the *tenuissimum* clade (Fig. 3B).

### Evaluation of lipolytic activity of selected fungi through the qualitative determination of rhodamine B

**Figure 4.**
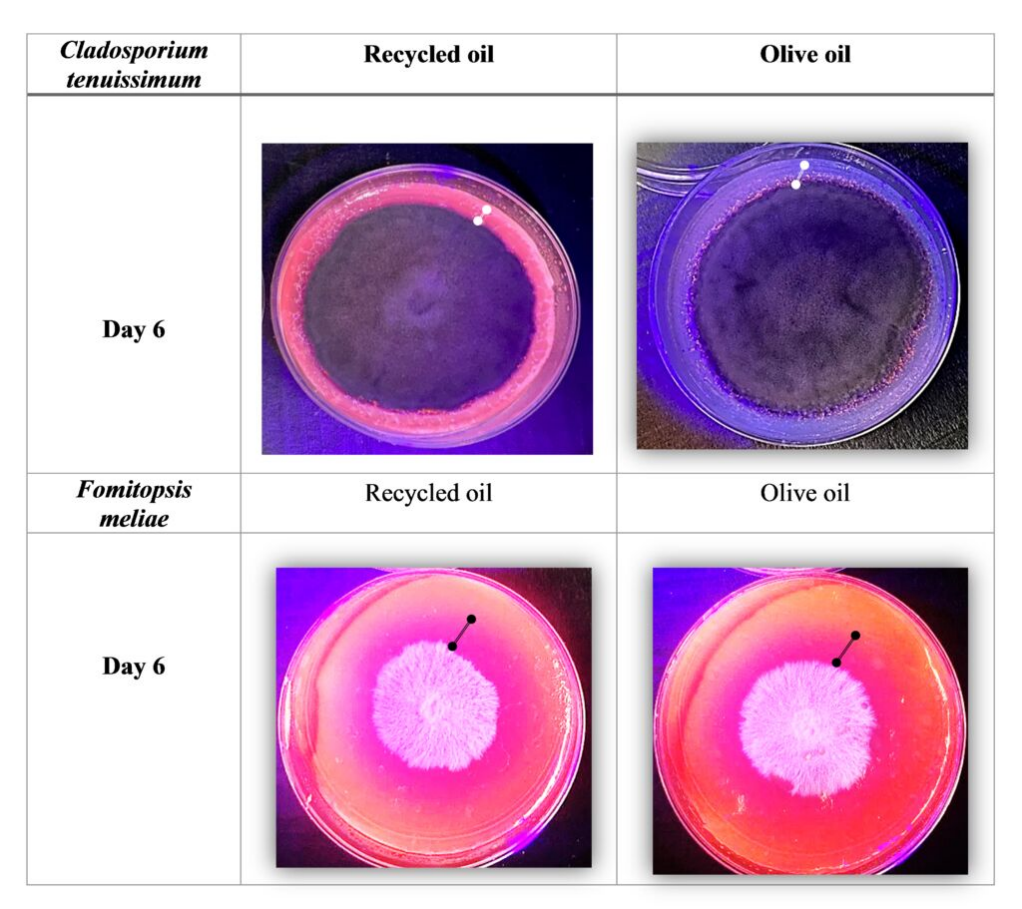
Qualitative assay with rhodamine B to determine the lipolytic activity of filamentous fungi.

For the determination of qualitative enzymatic activity with rhodamine B, it was observed that there were no considerable variations between the isolated strains, the plates with the highest fluorescence emitted corresponding to the medium in which *Fomitopsismeliae* was found, since the halo of illumination that is presented is evident. Under UV light, indicating a greater dimension of lipolytic capacity, this strain showed similar responses in both tests with different substrates, suggesting the feasibility of using olive or recycled oil interchangeably, while, for *Cladosporiumtenuissimum*, there was a lower presence of haloes of fluorescences on both substrates (Fig. 4).

The combination with rhodamine B is a colourimetric method, in which this compound binds to fatty acids and mono-and diglycerides and develops fluorescence under ultraviolet (UV) light. This assay is based on the interaction of the cationic form of rhodamine B with the anionic form of fatty acids, leading to the formation of complexes whose stability and fluorescence intensity is proportionally inverse to the length of the fatty acid chain (Mendes et al. 2019).

The results obtained are related to the living conditions of each filamentous fungus evaluated; since *Cladosporiumtenuissimum* is very demanding in its living conditions to promote its propagation, it requires a substrate with at least 1% sodium chloride (NaCl) under aerobic conditions, unlike of *Fomitopsismeliae* that corresponds to a Brown rot basidiomycetes where its ideal life condition corresponds to conditions of high humidity and preferably anaerobic processes, as it is a facultative microorganism with high metabolic malleability (Nam et al. 2015,Polanco-Florián et al. 2020).

The recognition of filamentous fungi with hydrolytic enzymatic activity suggests that they are preferred sources for the industry, due to superior metabolic versatility (Su et al. 2012). Fungal enzymes are used in biofuel production processes, bioremediation of organic contaminants, for detergent production, antibiotic production, bleaching of textile fabrics, juice clarification, removal of leather aromas and paper production (El-Sawah et al. 2021,Füting et al. 2021,Hyde et al. 2019,Kirk et al. 2002,Meyer et al. 2020,Molina-Gutiérrez et al. 2021,Niyonzima 2020, Singh et al. 2021, Sudeep et al. 2020, Syedd-León et al. 2022).

### Determination of enzyme activity (lipolytic)

After subjecting the fungus to olive and residual oil, it was identified that the highest concentration of crude protein was for *Fomitopsismeliae* with 24.79 mg/ml in recycled oil, followed by 13.60 mg/ml in olive oil, thus also observing the higher enzymatic activity (0.61 U/l) in this fungus when found in recycled oil (Table 3). These results coincide with those identified in the evaluation with rhodamine (Fig. 4).

The results agree with what was established by Mendes et al. (2019) who mention that the success in obtaining enzymes with hydrolytic capacity depends on the optimisation in culture conditions, necessary to achieve economic viability. Given that, *Fomitopsismeliae* is a basidium that is widely distributed, presenting greater adaptation in humid environments, facultative anaerobes and a high concentration of carbon structures, unlike *Cladosporiumtenuissimum* that requires low saline concentrations and aeration; therefore, oily substrates represent a problem suitable environment for its propagation.

Fungal species that have been described with biotechnological potential are *Rhizopus* sp., *Mucor* sp., *Geotrichum* sp., *Penicillium* sp., *Aspergillusniger*, *Aspergillus* sp., *Penicilliumchrysogenum*, *Rhizomucormiehei*, *Yarrowialipolytica*, *Fusarium* sp., *Neurospora* sp., amongst others. These species are specimens that present continuous and septate hyphae, with endogenous spores of the sporangium type and asci (Adamczak and Bednarski 2004, Carvalho et al. 2005, Cesário et al. 2021,Hita et al. 2009,Kumar and Negi 2015).

## Conclusions

The isolation and taxonomic and molecular identification of *Cladosporiumtenuissimum* and *Fomitopsismeliae* provide evidence of two native species that develop in used oil, demonstrating the presence of filamentous fungi in different organic environments. These microorganisms belong to strains adapted to the conditions typical of these residual oils, with Ascomycota and Basidiomycota domains, capable of developing in inhospitable niches lacking in nutrients that cells of other organisms probably cannot assimilate. The identification of these fungal strains contributes to the knowledge of this type of microorganism, opening the possibility of future studies on their metabolic diversity, in addition to the opportunity to study strains that have not been evaluated and that have biotechnological potential.

## Figures and Tables

**Table 1.** Physicochemical properties of recycled oil.

**Property**	**Unit**	**Value**
Relative density (15°C)	-------	0.92 ± 0.05
Viscosity (40°C)	mm²/s	60.95 ± 0.01
Humidity and volatile matter	% volume	0.55 ± 0.08

**Table 2.** Identification parameters of filamentous fungi, based on the ITS sequence.

**Sample**	**Sequence (ITS) length**		**Closest Blast match**	**Identity (%)**	**E-value**	**Query cover**
**Fungus with brown colour colonies**	546 bp	*Cladosporiumtenuissimum* (MF473304.1)		100%	0.0	100%
**Fungus with white colour colonies**	662 bp	*Fomitopsismeliae* (MW221272.1)		99.55%	0.0	100%

**Table 3.** Qualitative assay with rhodamine B to determine the lipolytic activity of filamentous fungi.

Fungi	Day	Concentration of crude protein generated by the fungus in olive oil (mg/ml)	Concentration of crude protein generated by the fungus in recycled oil (mg/ml)	Enzymatic activity [EA](U/l) in olive oil	Enzymatic activity [EA](U/l) in recycled oil
*Cladosporiumtenuissimum*	6	0.27 ± 0.00^Ab*^	0.0^Ab^	0.04 ± 0.01^b^	0.0^b^
*Fomitopsismeliae*	6	13.60 ± 0.05^Ba^	24.79 ± 0.62^Aa^	0.52 ± 0.01^Ba^	0.61 ± 0.02^A^ª
